# Synthesis, Crystallography, and Anti-Leukemic Activity of the Amino Adducts of Dehydroleucodine

**DOI:** 10.3390/molecules25204825

**Published:** 2020-10-20

**Authors:** Paola E. Ordóñez, David E. Mery, Krishan K. Sharma, Saumyadip Nemu, William F. Reynolds, Raul G. Enriquez, Darcy C. Burns, Omar Malagón, Darin E. Jones, Monica L. Guzman, Cesar M. Compadre

**Affiliations:** 1 Department of Pharmaceutical Sciences, University of Arkansas for Medical Sciences, Little Rock, AR 72205, USA; pordonez@yachaytech.edu.ec (P.E.O.); DMery@uams.edu (D.E.M.); sxnemu@ualr.edu (S.N.); enriquezhabib@gmail.com (R.G.E.); DEJones@uams.edu (D.E.J.); 2 School of Chemical Sciences and Engineering, Yachay Tech University, Urcuquí 100119, Ecuador; 3 Division of Hematology/Oncology, Department of Medicine, Weill Cornell Medical College, New York, NY 10065, USA; krishankumarsharma1@gmail.com; 4 Department of Chemistry, University of Toronto, Toronto, ON M5S 3H6, Canada; wreynold@chem.utoronto.ca (W.F.R.); darcy.burns@utoronto.ca (D.C.B.); 5 Instituto de Química, Universidad Nacional Autónoma de México, México 04510, DF, Mexico; 6 Departamento de Química, Universidad Técnica Particular de Loja, Loja 110107, Ecuador; omalagon@utpl.edu.ec

**Keywords:** dehydroleucodine, anti-leukemic activity, sesquiterpene lactone, amino adducts

## Abstract

Dehydroleucodine is a bioactive sesquiterpene lactone. Herein, four dehydroleucodine amino derivatives were synthesized using the amines proline, piperidine, morpholine, and tyramine, and spectroscopic methods and single-crystal X-ray diffraction unambiguously established their structures. The cytotoxic activity of these compounds was evaluated against eight acute myeloid leukemia cell lines, and their toxicity to peripheral blood mononuclear cells was also determined. The proline adduct was the most active compound, it showed anti-leukemic activity, upregulated heme oxygenase 1 (HMOX1) and the primary stress-inducible isoform of the heath shock 70 kDa protein 1 (HSPA1A), and downregulated NFkB1 transcription, it was also found to be about 270 times more water soluble than dehydroleucodine.

## 1. Introduction

Sesquiterpene lactones (SLs) are a large and diverse group of biologically active plant chemicals, mainly distributed in the Asteraceae family. Parthenolide, one of the most widely studied SLs, is extracted from the medicinal plant feverfew, *Tanacetum parthenium* L., a herb that has been used to treat migraines and arthritis for centuries [1]. Parthenolide has been evaluated in different biological systems, including the inhibition of acute myeloid leukemia (AML) stem and progenitor cells [1]. Other SLs of the guaianolide type have also been shown to have activity against AML [2]. We previously reported that dehydroleucodine (DHL), a guaianolide SL isolated from the Ecuadorian medicinal plant *Gynoxys verrucosa* Wedd., shows activity against several AML cell lines, exhibits reduced toxicity to normal blood cells relative to AML cell lines, and shows activity against human leukemia cells isolated from patient samples [3]. The postulated general mechanism of the action of SLs is that the exocyclic methylene group in the lactone ring undergoes a nucleophilic attack by endogenous nucleophiles, such as a thiol in the cysteine residue of the protein, leading to the formation of covalent adducts [4,5].

In general, SLs lack water solubility, resulting in poor drug-like properties. In order to mitigate this limitation, an amino-prodrug strategy has been reported. This strategy utilizes the masking of exocyclic methylene in the lactone ring by reaction with primary or secondary amines. It has been proposed that in vivo, the exocyclic double bond is regenerated [6]. Several amino analogues of parthenolide have shown anti-leukemic activity [7], including dimethylamino parthenolide, raising the interest in using it as a potentially clinically effective anticancer compound alone or in combination with other anticancer compounds [8,9].

SLs have shown intracellular effects in various cancer cell lines, including inhibition of nuclear factor-κB (NF-κB), inhibition of sarco/endoplasmic reticulum calcium ATPase (SERCA) pump, iron-dependent free radical generation, blocking of the signal transducer and activator of transcription 3 (Stat3), p53 activation, and COX-2 inhibition [2,10,11,12]. The molecular mechanisms of action of compound **1** for AML-specific cell death was found to be the inhibition of NF-κB and the upregulation of heme oxygenase 1 (HMOX1) and the primary stress-inducible isoform of Hsp70 (HSPA1A) [3].

NF-κB has an important role in the regulation of cell cycle progression, proliferation, and apoptosis. It can be formed by homodimers and heterodimers of a p50 and a p65 subunit. This protein is found in the cytoplasm of every cell in its inactive state, in which it is normally bound to inhibitory proteins, including the inhibitor of κB (ΙκB). When cells are exposed to invaders or stressors, it travels to the nucleus and binds with DNA to turn certain genes on or off. On activation, NF-κB regulates the expression of approximately 400 different genes [13]. Meanwhile, dysregulation of NF-κB can generate several diseases, including inflammation and cancer [14].

Experimental evidence suggest that SLs react by a Michael addition mechanism with the cys-38 residue in the p65 subunit of NF-κB, preventing it from binding to DNA [14,15,16].

Herein, we report the synthesis and chemical characterization of four amino derivatives of dehydroleucodine, as well as their evaluation against several AML cell lines.

## 2. Results and Discussion

### 2.1. Chemistry

**Scheme 1:**
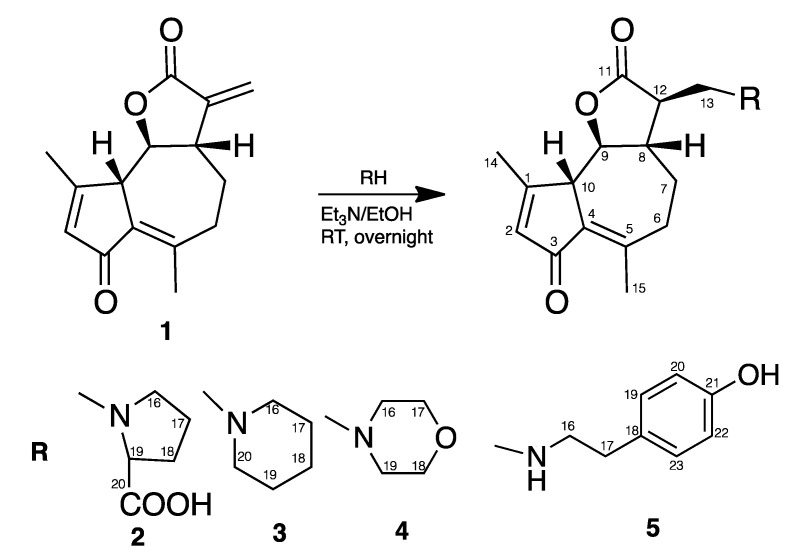
Synthesis of the amino derivatives of compound **1**.

Compound **1** was isolated from *G. verrucosa* following the procedure previously described [3]. Four amino adducts of compound (**1**), namely, dehydroleucodine-proline (**2**), dehydroleucodine-piperidine (**3**), dehydroleucodine-morpholine (**4**), and dehydroleucodine-tyramine (**5**), were synthesized by stirring a solution of **1** at room temperature (RT) with the appropriate amine in a single-step reaction using an adaption of a previously reported procedure (Scheme 1). In all of the cases, single diastereoisomeric products were obtained after purification (60–91% yield).

**Figure 1:**
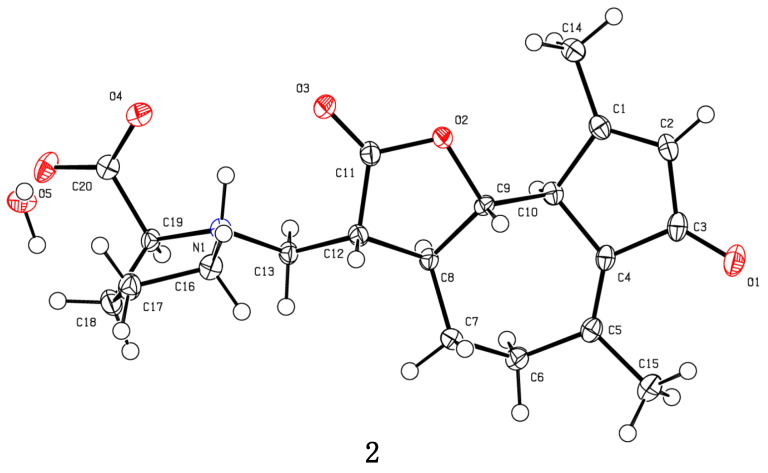
Three dimensional (3D)-ORTEP (name of computer program) projections of the X-ray crystal structure of compound **2** with 50% probability of ellipsoids.

Compound **2** is a white powder with a molecular formula of C_20_H_25_NO_5_ based on high-resolution mass spectroscopy (HRMS) (*m/z* 360.1823 [M + H]^+^) data. The IR (attenuated total reflectance (ATR)) spectrum has absorption bands (*v*_max_) at 3510 (COO–H st), 1760 (C=O st γ lactone), 1670 (α–β unsaturated C=O), 1645, 1633, and 1613 (C=C st) cm^−1^. Complete ^1^H and ^13^C NMR data of **2** are provided in Table 1. The assignment of the ^1^H and ^13^C NMR resonances was accomplished using two-dimensional (2D) correlation spectroscopy (COSY), heteronuclear multiple quantum coherence (HMQC), rotating-frame Overhauser effect spectroscopy (ROESY), and heteronuclear multiple-bond correlation (HMBC) experiments. The proton and carbon resonances appeared at nearly the same chemical shifts and with similar multiplicities as dehydroleucodine, except for the δ 2.25 signal for H-12 and the signals corresponding to H-13, which appeared as a doublet at δ 1.27 in the ^1^H NMR spectrum, suggesting the presence of a C–H bond on the lactone ring. This result is also consistent with the changes in the ^13^C NMR spectrum. Thus, a large (*anti*) H-8/H-12 ^3^J coupling, as well as a ROESY peak between H-12 and H-9, were observed, indicating that these hydrogens lie on the same molecular face. In addition, a ROESY peak between H-12 and H-8 indicates that these hydrogens also lie on the same face. Finally, the structure of compound **2** was confirmed by X-ray crystallographic analysis, and an Oak Ridge thermal ellipsoid plot ORTEP perspective is shown in Figure 1.

The crystal structure of **2** shows that the H atom of the carboxyl group migrated to the N atom in the molecule. The differential electron density maps between compounds **2** and **1** clearly show the location of this H atom, as well as its absence on the COO functional group. Additionally, the H atoms of an associated water molecule were found through electron density map differences.

Compound **3** is a pale yellow amorphous solid with a molecular formula of C_20_H_27_NO_3_ based on HRMS data (*m/z* 330.2075 [M + H]^+^). The UV spectrum in methanol (MeOH) shows two peaks at λ (log ϵ) 204 (3.66) nm and 256 (4.14) nm. The IR (ATR) spectrum shows bands at 2936 (C–H st), 1773 (C=O st γ lactone), 1683 (α,β unsaturated C=O), 1617, and 1637 (C=C st) cm^−1^. The ^1^H and ^13^C NMR chemical shifts are shown in Table 1. Assignments of all of the proton and carbon resonances were achieved by using the 2D NMR, COSY, ROESY, and HMBC techniques.

**Figure 2:**
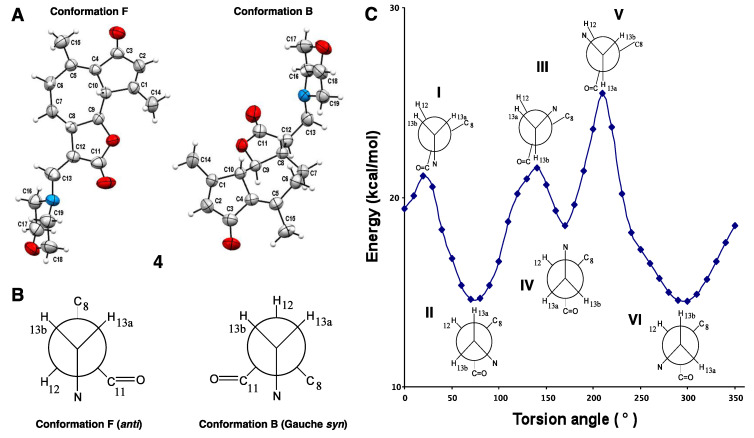
(**A**) 3D-ORTEP projections of the X-ray crystal structures of compound **4** with 50% probability of ellipsoids. (**B**) Conformations of compound **4**. (**C**) Conformational analysis of compound **4**, the lowest energy conformations II and VI correspond to the conformations observed in the X-ray crystal (**A**).

Compound **4** comprises colorless crystals with a mp of 158–159 °C and a molecular formula of C_19_H_25_NO_4_ based on HRMS data (*m*/*z* 332.1848 [M + H]^+^). The UV spectrum (MeOH) shows two peaks at λ (log ϵ) 204 (3.99) nm and 256 (4.28) nm. The IR (ATR) spectrum shows bands at 2930 (C–H st), 1770 (C=O st γ lactone), 1680 (α-β unsaturated C=O), 1640, 1610 (C=C st), and 1110 (C–O–C) cm^−1^. The ^1^H and ^13^C NMR assignments are given in Table 1 and were assigned by the COSY, ROESY, HMQC, and HMBC experiments. The relative configuration of compound **4** was determined by analysis of its nuclear Overhauser enhancement spectroscopy (NOESY) data. A nuclear Overhauser effect (NOE) correlation was observed between the H-8 and H-13 methylene protons, but not with H-12. H-8 shows three large (10–12 Hz) vicinal couplings to H-9, H-12, and one of the H-7 (δ 1.36) protons, indicating that it is anti to H-9, H-12, and a H-7 proton. H-9 shows strong NOE correlations with H-12 and the δ 1.36 proton. Thus, H-8 and H-12 are on opposite faces of the molecule. Ultimately, the structure of **4** was confirmed by X-ray crystallographic analysis, as shown in Figure 2A.

The crystal unit cell of **4** is composed of two different conformers, as shown in Figure 2A; one conformation is *trans* at the C-13-N and C-H-12 bonds, and the other is gauche *cis* (Figure 2B). A systematic conformational analysis of the rotation around the bond between C-12 and C-13 of compound **4** showed three minima and three maxima (Figure 2C). The lowest two minima, conformations II and VI with energies of 14.60 and 14.61 kcal/mol, respectively, correspond to the conformations shown in the crystal structure unit cell. The three maxima, conformations I, III, and V with energies of 21.00, 21.50, and 25.40 kcal/mol, respectively, correspond to the eclipsed conformations.

Compound **5** is a white amorphous powder with a molecular formula C_23_H_27_NO_4_, mw = 881.47, mp = 90–95 °C. The IR (ATR) spectrum shows absorption bands at 3270 indicating the presence of hydroxyl groups, 2920 C-H aromatic, 2850 C-H st, 1760 C=O st γ lactone, 1680 α-β unsaturated C=O and 1610 C=C st. The EI spectrum showed one main fragment at *m*/*z* 244 (100%). The ^1^H and ^13^C NMR chemical shifts are provided in Table 1. Assignments of proton and carbon resonances were completed by using 2D NMR, COSY and HMBC techniques. The J8–12~13Hz, indicates that H-8 and H-12 are anti and therefore on opposite faces. In the nuclear Overhauser effect (NOE) experiment on compound **5**, a NOE correlation was observed between H-9 and H-12 protons indicating that they are on the same face, this is supported by moderate NOESY correlations between H-8 and both H-13 protons.

### 2.2. Anti-Leukemic Activity

Table 2 shows the cytotoxic activity of the amino derivatives against eight AML leukemia cell lines after 48 h of treatment. Compounds **2**–**4** showed in vitro cytotoxicity at a more moderate level than compound **1**. Compound **2** was the most potent derivative and compound **5** was found to be inactive.

**Figure 3:**
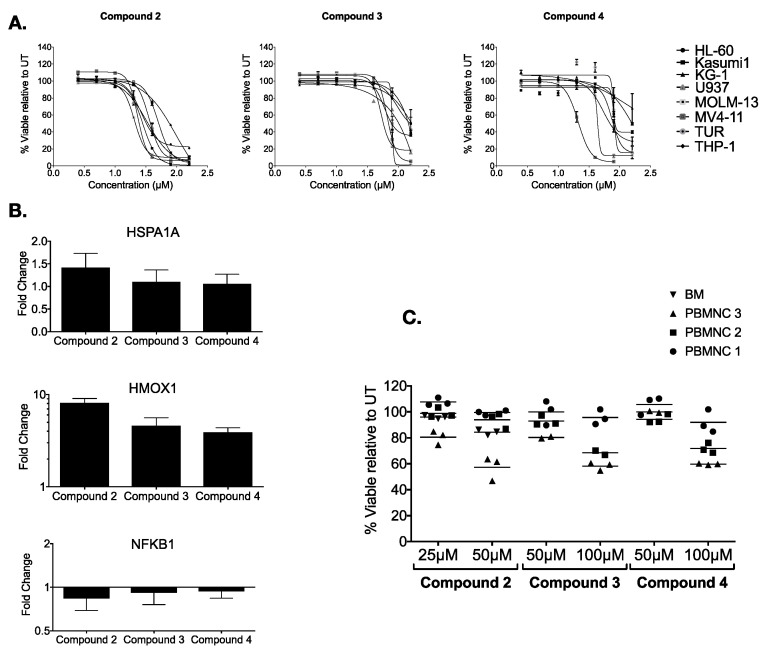
(**A**) Cytotoxic effects of compounds **2**–**4** against eight acute myeloid leukemia (AML) cell lines. Viability of the cells after 48 h of treatment at different concentrations, from 1.25 to 160 μM. (**B**) Compounds **2**–**4** induce heme oxygenase 1 (HMOX1) and HSPA1A and downregulate NF-κB. Graphic B represents the fold changes of HSPA1A, HMOX1, and NFkB1A gene expression in MOLM-13 cells. Lines represent the mean for each specimen, with error bars representing the standard deviation (SD). The fold changes were calculated by the delta-delta Ct method. (**C**) The viability of normal cells after treatment with **2**, **3**, and **4**. Bone marrow and peripheral blood mononuclear cells were isolated from blood purchased form the New York Blood Center, and treated for 48 h with 25 µM or 50 µM of compound **2**, and 50 µM or 100 µM of compounds **3** or **4**. The average viability was evaluated by annexin V and 7AAD staining using flow cytometry. Each line represents a distinct sample with viabilities calculated relative to an untreated control.

In addition, we evaluated the intracellular effects upon treatment with the amino derivatives **2**–**4** using quantitative PCR (Figure 3B). Leukemia cells were treated with 20 µM of each test compound for 6 h before RNA extraction. HMOX1 and HSPA1A were measured to determine the activation of an oxidative stress response upon drug treatment. Previously, we reported that compound **1** upregulated HMOX1 and HSPA1A, suggesting that this compound activates oxidative stress responses [3]. We performed the same experiment for the amino derivatives of compound **1**; the results show that compounds **2**–**4** moderately upregulated HMOX1 and HSPA1A. Interestingly, compound **2** displayed a higher upregulation of HMOX1 and HSPA1A compared to **3** and **4**, and it also displayed greater cytotoxic effects on the AML cell lines, as shown in Table 2 and Figure 3A. The NF-κB transcript levels were measured upon treatment with compounds **2**–**4** (Figure 3B), and in all cases we observed a downregulation of NFkB1 transcription.

The activity of compounds **2**–**4** was also tested on normal bone marrow (*n* = 1) and peripheral blood (*n* = 3) mononuclear cells. Figure 3C displays the viability of the normal samples after 48 h of treatment with the test compounds. At 25 µM, compound **2** treated normal cells showed an average viability of 90%; at 50 µM, cells treated with compounds **2**, **3**, or **4** showed an average viability of 78, 80, and 79%, respectively; at 100 µM, cells treated with compounds **3** or **4** showed an average viability of 74 and 73%, respectively. Comparison of these results with the average EC_50_ values for the leukemia cell lines (Table 2) shows that the tested compounds exhibited less toxicity against normal cells than against leukemia cell lines.

The four amino derivatives of dehydroleucodine were synthetized by a regioselective and stereoselective conjugate addition reaction and tested against eight AML cell lines. All of the derivatives showed less activity compared to the parent compound. Among the derivatives, DHL-proline (**2**) showed the most activity and displayed higher upregulation of HMOX1 and HSPA1A.

The strategy of synthetizing amino derivatives has previously been applied to different sesquiterpene lactones, including helenalin, ambrosin, costunolide, alantolactone, and parthenolide [6]. In some cases, the amino derivatives of SLs showed similar activity to their respective parent compounds, for example, the pyrrolidine and piperidine analogues of ambrosin, the diethylamine derivatives of alantolactone and isoalantolactone, the dimethylamine, pyrrolidine, and piperidine adducts of custonolide, and the proline amino derivative of dehydrocostus lactone [6]. However, as has been reported for helenalin [6], and as we have shown in this paper for dehydroleucodine, it is clear that in some cases, the amino derivatives of these SLs can be less active than their parent compounds.

The solubility (±SD) of the most active derivative, compound **2**, was 64.62 ± 5.47 mg/mL when measured in buffer at pH 7.4, and the solubility of compound **1** was 0.24 ± 0.02 mg/mL when measured in the same conditions. These results show that the proline derivative is about 270 times more water soluble than the parent compound.

Compound **1** has potent activity against leukemia cell lines and leukemia primary cells, is less toxic to primary mononuclear cells than to cancer cells [3], is very abundant in nature, easy to isolate and relatively stable. We have also determined, experimentally, that compound **1** has an octanol/water Log P (±SD) of 2.33 ± 0.16, which is well within the range of the optimum partition for cell permeation (Log P = 2–3.5) [17]. Unfortunately, compound **1**, has very limited water solubility and that limits its applicability, in this context the high solubility of the proline adduct **2** bodes well for its potential as dehydroleucodine prodrug.

## 3. Materials and Methods

### 3.1. General Experimental Procedures

Melting points were measured with a capillary melting point apparatus, and the values were uncorrected. Optical rotations were measured on an Autpol III (Rudolph) polarimeter. The UV spectra were recorded on a Hewlett–Packard 8452A diode array spectrophotometer, while the IR spectra were measured using a Magna IR 550 spectrometer with attenuated total reflectance (ATR). NMR spectra were recorded in CDCl_3_ at 25 °C on a Varian Unity NMR spectrometer equipped with a Nalorac 3 mm inverse detection probe with a z-axis gradient coil, operating at 500 MHz for ^1^H and 125 MHz for ^13^C spectra. Each sample consisted of approximately 5 mg of the desired compound dissolved in 1 mL CDCl_3_ containing TMS as an internal reference. All of the one-dimensional (1D) and 2D (i.e., gradient COSY, ROESY, gradient HSQC, and gradient HMBC) spectra were acquired and processed with standard Varian software. Flash chromatography was performed using a Teledyne ISCO CombiFlash Companion Automated Flash Chromatography system with pre-packed silica gel columns. Column chromatography was performed using 60–230 mesh silica gel. All of the reagents were used as purchased.

### 3.2. General Procedure for the Preparation of Amino Adducts

A solution of **1** (0.22 mmol) in EtOH (6 mL) was treated with the corresponding amines (4.4 mmol) in the presence of Et_3_N, and the mixture was stirred at room temperature overnight. The reaction mixture was evaporated under reduced pressure, and the residue was further purified by silica gel flash chromatography to yield products **4**–**5**. All of the reactions were performed under a dry nitrogen atmosphere.

Dehydroleucodine-proline **2**. Colorless crystals; mp 160–165 °C, [α]_25D_ +13.89 (c 1.8, methylene chloride); UV (MeOH) λ (log ϵ) 204 (3.60) nm and 256 (4.16) nm; IR (ATR) (vmax) 3510, 1760, 1670, 1645, 1633, and 1613 cm^−1^; ^1^H and ^13^C NMR (CDCl_3_, 500 and 125 MHz), see Table 1; HRMS (ESI) observed C_20_H_26_NO_5_ 360.1823 [MH^+^] requires 310.1811.

Dehydroleucodine-piperidine **3**. Pale yellow solid; [α]_25D_ +29.59 (c 1.96, MeOH); UV (MeOH) λ (log ϵ) 204 (3.60) nm and 256 (4.14) nm; IR (ATR) (vmax) 2936, 1773, 1683, 1617, and 1637 cm^−1^; ^1^H and ^13^C NMR (CDCl_3_, 500 and 125 MHz), see Table 1; HRMS (ESI) observed C_20_H_28_NO_3_ 330.2075 [MH^+^] requires 330.2069.

Dehydroleucodine-morpholine **4**. Colorless crystals; mp 158–159 °C; [α]^25D^ +30.75 (c 3.09, MeOH); UV (MeOH) λ (log ϵ) 204 (3.99) nm and 256 (4.28) nm; IR (ATR) (vmax) 2930, 1770, 1680, 1640, 1610, and 1110 cm^−1^; ^1^H and ^13^C NMR (CDCl_3_, 500 and 125 MHz), see Table 1; HRMS (ESI) observed C_19_H_26_NO_4_ 332.1848 [MH^+^] requires 332.1862.

Dehydroleucodine-tyramine **5**. White powder; mp 90–95 °C; IR (ATR) (vmax) 3270, 2920, 2850, 1760, 1680, and 1610 cm^−1^; ^1^H and ^13^C NMR (CDCl_3_, 500 and 125 MHz), see Table 1; MS (EI) observed C_23_H_27_NO_4_ 881.47 [MH^+^].

Single-crystal X-ray diffraction analysis and crystallographic data for compounds **2** and **4**. Crystallographic data for the structures were deposited in the Cambridge Crystallographic Data Centre, and the deposition number is indicated below. The data can be obtained free of charge at www.ccdc.cam.ac.uk (or from the Cambridge Crystallographic Data Centre, 12 Union Road, Cambridge CB2 1EZ, U.K.; fax (+44) 1223-336-033 or email desposit@ccdc.cam.ac.uk).

Crystallographic data of **2**. Crystallization from ethyl acetate yielded colorless crystals of **2**. The X-ray data were collected on a Bruker-Nonius X8 Proteum CCD diffractometer using CuKα radiation. The structures were solved using SHELXT and were refined using SHELXL. Molecular fragment editing was performed using the XP program of SHELXTL [18]. Crystal data: C_20_H_25_NO_5_.H_2_O, Mw = 377.43 g mol^−1^, space group P21 21 21, a = 5.9468(2) Å, b = 7.3005(2) Å, c = 42.5831(13) Å, α = β = γ = 90.00, V = 1848.73(10) Å^3^, T = 90 K, Z = 4, Dc = 1.356 g/cm^3^, R^1^ = 0.0337 (wR2 = 0.0883); Flack parameter = 0.07(5). Deposition number: CCDC 1050743.

Crystallographic data of **4**. Crystallization from an ethyl acetate–hexane mixture (2:1) yielded colorless crystals of **4**. The X-ray data were collected on a Bruker Smart APEX AXS CCD diffractometer with Mo-Kα radiation at room temperature. Crystal data: C_19_H_25_NO_4_, Mw = 331.40 g mol^−1^, size 0.40 0.30 × 0.20 mm, monoclinic, space group P21, a = 10.7660(11) Å, b = 12.8159(13) Å, c = 12.6068(13) Å, α = γ = 90°, β = 96.39°, V = 1728.6(3) Å^3^, Z = 4, T = 298(2) K, Dc = 1.273 mg/m^3^, λ = 0.71073 Å, R^1^ = 0.0372 for 14,372 observations with I > 2σ(I), R^1^ = 0.0501 (wR2 = 0.0728). Deposition number: CCDC 1050742.

### 3.3. Conformational Analysis

Compound **4** was analyzed using the grid search routine implemented in SYBYLX2.1.1 (Certara, St. Louis, MO, USA). This routine performs a molecular mechanics minimization for every conformer in the systematic search. These conformers were obtained by performing systematic torsion angle changes around the C-12 and C-13 single bond in 10-degree increments using the Steepest-Descendent method for energy minimization, with 1000 iterations and a gradient of 0.05. The starting geometries were optimized using MOPAC (Name of a program) electrostatic charges and the TRIPOS (Name of a program) force field, with a dielectric function distance and 8.0 as the non-bonded cutoff.

### 3.4. Biological Activity

Cell lines and cell culture. Bone marrow and peripheral blood samples were obtained from healthy donors with informed consent under WCMC IRB 0909010629 approval. Mononuclear cells were isolated from the samples using Ficoll-Paque (Pharmacia Biotech, Piscataway, NJ USA) density gradient separation. Cells were cultured in serum-free medium (SFM) supplemented with cytokines (50 ng/mL rhFLT-3 ligand, 50 ng/mL rhSCF, 20 ng/mL rhIL-3, and 20 ng/mL rhIL-6) for 1 h before the addition of drugs. The HL-60 (purchased 9/2010, ATCC), Kasumi-1 (purchased 4/2011, ATCC), KG-1 (purchased 9/2010, ATCC), MOLM-13 (kind gift form G. Chiosis-MSKCC 7/2010, 2/2014 authenticated; biosynthesis), MV4-11 (purchased 9/2010, ATCC), THP-1 (purchased 9/2010, ATCC), TUR (purchased 1/2010, ATCC), and U937 (purchased 12/2009, ATCC) cell lines were cultured in Iscove’s Modified Dulbecco’s Medium (IMDM; Life Technologies) supplemented with 10–20% fetal bovine serum (FBS), according to the culture conditions indicated by ATCC, and with 1% penicillin/streptomycin (Pen/Strep; Life Technologies) at 37 °C and 5% CO_2_.

Flow cytometry and cytotoxicity assays. The cells were seeded into 96-well plates and were maintained at a concentration of 0.5 million cells per milliliter. The samples were treated with compounds **2**–**5** at varying concentrations in triplicate. Cell counting for these experiments was performed using the Invitrogen Cell Countess system with Trypan Blue stain. Cell viability was determined after incubating for 48 h. The cells were stained with annexin V–fluorescein isothiocyanate (FITC; Molecular Probes-Invitrogen) or phycoerythrin (PE; Molecular Probes-Invitrogen) and 7-aminoactinomycin (7-AAD; Molecular Probes-Invitrogen) to detect phosphatidylserine exposition and cell permeability, respectively. At least 50,000 events were recorded per condition on either an LSR-II or an LSR-Fortessa flow cytometer (BD Biosciences). Data analysis was conducted using FlowJo 9.6 software for Mac OS X (TreeStar). Cells that were negative for annexin V and 7-AAD were scored as viable. Analyses and graphs were produced using the GraphPad Prism software.

RNA extraction and quantitative PCR. MOLM-13 cells were seeded at 0.5 million cells per milliliter. Cells were treated for 6 h with 20 µM of the tested compounds, and then collected. Total RNA was extracted using the Qiashredder and Qiagen Rneasy Mini kits (Quiagen) according to the manufacturer’s instructions. Experiments were performed in triplicate. Real-time PCR was performed using the Taqman RNA to CT 1-Step kit (Applied Biosystems). The thermal cycling conditions were one RT step (48 °C, 15 min), enzyme activation (95 °C, 10 min), 40 cycles of denaturing (95 °C, 15 s), and annealing/extension (60 °C, 1 min). Quantitative PCR was performed using probes for HMOX1 (Hs01110251_m1), HSPA1A (Hs00359163_s1), and NFkB1 (Hs00765730_m1). GADPH (Hs02758991_g1) was used as the housekeeping gene, an internal control to normalize the variability in expression levels. All of the probes were provided by Applied Biosystems. Single-plex real-time PCR was performed in triplicate in a StepOne Plus Real-Time PCR system (Applied Biosystems) and analyzed with the StepOne Software. Fold change was calculated using the 2-ddCt method described by Livak and Schmittgen [19].

### 3.5. Determination of Partition Coefficient and Solublity

The experimental octanol/water partition coefficient of compound **1** was determined by quadruplicate measurements at two concentrations (1 and 0.25 mg compound/mL of octanol) using the shake-flask method [20], in which the compound was fractionated between octanol (1 mL) and 100 mL of deionized water into glass bottles of 150 mL. Both phases were pre-saturated before the determination. The glass bottles were placed in a shaker for 1 h and then centrifuged for 30 min at 20 °C and 5000 rpm. For the quantitation 1 mL aliquotes from the aqueous phase were evaporated under nitrogen the residue was then reconstituted in a 200 µL glass insert with methylene chloride.

To determine the solubility compounds **1** and **2**, supersaturated mixtures of the compounds, in phosphate buffered (11.9 mM) saline (pH 7.4) were sonicated (30 min at 25 °C), vortexed (90 min at 25 °C), and centrifuged (30 min at 13,600 G). Aliquots of the supernatants were passed through a syringe filter, and the amounts of compounds **1** and **2** were determined. The experiments were conducted in triplicate.

The analytical determination of the partition coefficient compound **1** and the solubility of compounds **1** and **2** were performed using single ion monitoring gas chromatography/mass spectrometry (GC/MS) with an Agilent 5975 GC/MSD instrument. The solubility determinations were conducted using a 30 m DB-5MS column (0.250 mm 10 μm, J&W, Folson, CA, USA) with a helium flow rate of 1.8 mL/min, the injector temperature was 200 °C, the transfer line was maintained at 280 °C, and column temperature was maintained at 170 °C for 1 min followed by a gradient of 35 °C/min to 270 °C, and then a gradient of 100 °C/min to 300 °C. The mass spectrometer conditions were electron impact, ion source temperature 230 °C, and ionization voltage 70 eV. The injection volume was 0.2 uL for compound **1**, and 0.5 uL for compound **2**. A calibration curve for the analytical method was developed with five points, plotting the average ratio of the peak area of the respective compound to the peak area of parthenin internal standard (R^2^ = 0.999).

The partition coefficient determination was conducted using a 15 m DB-1 column (0.250 mm 10 μm, J&W, Folson, CA, USA) with a helium flow rate of 1.8 mL/min, the injector temperature was 200 °C, the transfer line were maintained at 300 °C, the column temperature was maintained at 120 °C for 5 min followed by a gradient of 17 °C/min to 300 °C. The mass spectrometer conditions were electron impact, ion source temperature 200 °C, and ionization voltage 70 eV. A five-point calibration curve (R^2^ = 0.999) was used to calculate the concentration of **1** in the aqueous phase using calaxin as the internal standard. The milligrams of compound **1** in the octanol phase were then indirectly calculated and the concentrations of each compound in each phase were used to determine the partition coefficient. The average of the measurements is reported as the logarithm of the octanol/water partition coefficient (Log *P*).

## Figures and Tables

**Table 1:** NMR Spectroscopy Data (500 MHz, CDCl_3_) of compounds **2**–**5**.

	2				3					4				5			
Position	δ_H_	δ_C_	HMBC	COSY	δ_H_	δ_C_	HMBC	COSY	δ_H_		δ_C_	HMBC	COSY	δ_H_	δ_C_	HMBC	COSY
1		170.3				170.1					169.9				170.1		
2	6.17	135.8	196.4, 170.3, 131.8, 52.4, 19.9	3.58, 2.28	6.16	135.6	196.1, 170.1, 132.0, 52.8, 20.0	3.43, 2.29, 6.16	6.17 (q, 1.3)		135.5	196.0, 169.9, 131.9 52.4, 19.7	3.43 [2.29] ^a^	6.15 (bs)	135.7	196.1, 170.1, 131.4, 52.6, 20.0	[3.38], [2.26] ^a^
3		196.4				196.1					196.0			2.38 (m, 1H)	196.1		
4		131.7				132.0					131.9				131.4		
5		153.0				152.8					152.4				152.5		
6	2.46 2.33	37.4	153.0, 131.7, 52.9, 25.6, 21.7	2.33	2.43 2.30	37.9	152.8, 132, 26.5, 54.8, 37.9	2.20, 2.40, 2.43	2.43 (m) 2.30 (m)		37.4			2.36 (m) 2.28 (m)	37.6		2.28, 1.96, 1.32 2.36, 1.96, 1.32
7	2.08, 1.44	25.6	153.0, 84.6, 52.4, 37.4 153.0, 84.6, 52.4, 37.4	2.46, 2.33, 1.44 24.6, 2.33, 2.08	2.40 1.34	26.5		2.60	2.34 (m) 1.36 (m)		26.1	152.4, 84.0, 54.1, 37.4	2.43, 2.34, 2.30, 2.24	1.96 (m) 1.32 (m)	26.1	152.5, 84.9, 51.8, 37.6	2.36, 2.28 2.25, 1.32 2.36, 2.28, 2.25, 1.96
8	2.34	52.4	153.0, 131.7, 52.9, 25.6, 21.7		2.20	54.8	84.3, 52.8	2.40, 2.41, 3.60, 3.43, 2.79	2.24 (m, −12, 12, 10, 3)		54.1		3.62, 2.41, 2.34, 1.36	2.25 (m)	51.8		3.61, 2.40, 1.96, 1.32
9	3.75	84.6	173.0, 131.7, 52.4, 43.4, 29.2, 25.6, 24.2	3.50, 2.34	3.60	84.3	170.2, 132.0, 52.8, 20.0	6.16, 3.60, 3.43, 2.20, 2.29	3.62 (t, 10.1)		84.0	131.9, 43.7, 26.1	3.43, 2.24	3.61 (t, 10)	84.6	170.1, 131.4, 46.5, 26.1	3.38, 2.25
10	3.50	52.4	170.3, 131.7, 135.8, 84.6, 52.4	6.17, 3.75, [2.42] ^a^, [2.25] ^a^	3.42	52.8	196.1, 170.1, 152.8, 135.7, 132.0, 84.3, 54.8	3.60, 6.16, 3.43, 2.29, 2.41	3.43 (bd, 10)		52.4	196.0, 169.9, 152.4, 135.5, 131.7, 84.0, 54.1	6.17, 3.62, [2.43] ^a^, [2.29] ^a^	3.38 (d, 10)	52.6	196.1. 170.1, 152.5, 135.7, 131.4, 84.6, 51.8, 20.0	[6.15] ^a^,3.61, [2.41] ^a^, [2.26] ^a^
11		176.0				176.9					176.3				176.9		
12	2.76	43.4	176.0	3.41, 3.21, 2.34	2.41	44.0		2.20, 2.60, 2.79	2.41 (m)		43.7			2.40 (ddd)	46.5		3.00, 2.82, 2.25
13	3.41 3.21	52.8	176.0, 69.2, 54.7, 43.4 176.0, 54.7, 52.4, 43.4	3.21, 2.76 3.41, 2.76	2.79 2.60	58.2	176.9, 54.8,44.0 176.9, 54.8, 44.0	2.41, 2.20, 2.79, 2.40, 2.20	2.84 (dd, 13.2, 4.7) 2.63 (dd, 13.2, 7.4)		57.2	176.3, 54.1, 43.7 176.3, 54.1, 43.7	2.63, 2.41 2.84, 2.41	3.00 (dd) 2.82 (dd)	46.7	176.9, 51.8, 46.5	2.82, 2.40 3.00, 2.40
14	2.28	19.9	170.3, 135.8, 52.4	6.17, 3.50	2.29	20.0	170.2, 152.6, 135.6, 131.9, 52.8, 26.2	6.16, 3.43	2.29 (3H, dd, 1.3, 0.9)		19.7	169.9, 135.5, 52.4	[6.17] ^a^, [3.43] ^a^	2.26(s, 3H)	20.0	170.1, 13.7, 52.6	[3.38]
15	2.42	21.7	153.0, 131.7, 37.4	3.50	2.43	21.7		3.4	2.43 (3H, bs)		21.3	152.4, 131.9. 37.4	[3.43] ^a^	2.41(s, 3H)	21.8	152.5, 131.4, 37.6	[3.38]
16	3.66 2.80	54.7	69.2, 52.8, 29.4, 24.2 52.8, 29.4, 24.2	2.80, 2.0 3.66, 2.0	2.42 2.32	55.0		2.32, 2.60, 2.79 2.42, 2.60, 2.79	2.51 (2H, m) 2.43 (2H, m)		53.9	66.7, 53.9	2.43 2.51	2.85(m, 2H)	51.9	131.9, 46.7, 35.3	2.74
17	2.00 (2H)	24.2	69.2, 54.7, 29.4	3.66, 2.8	1.54	26.2	55.0, 26.2, 24.3	2.42, 1.43	3.69 (4H, m)		66.7	66.7, 53.9	2.51, 2.43	2.74(t, 2H, 9)	35.3	131.9, 130.0, 51.9	2.85
18	2.32 2.23	29.4	153.0, 131.7, 52.9, 25.6, 21.7		1.43	24.3	55.0, 26.2	1.54, 2.42	3.69 (4H, m)		66.7	66.7, 53.9	2.51, 2.43		131.9		
19	3.73	69.2	2.32, 2.23		1.54	26.2	55.0, 26.2, 24.3		2.51 (2H, m) 2.43 (2H, m)		53.9	66.7, 53.9	2.43 2.51	7.05(d, 2H, 8)	130.0	154.6, 130.0, 115.6, 35.3	6.84
20		173.0			2.42, 2.32	55.0		2.32, 2.60, 2.79 2.42, 2.60, 2.79						7.05(d, 2H, 8)	57.4	154.6, 131.9, 115.6	7.05
22														6.84(d, H, 8)	115.6	154.6, 131.9, 115.6	7.05
23														7.05(d, 2H, 8)	115.6	154.6, 130.0, 115.6, 35.3	6.84

^a^ Figures in square brackets denotate weak long-range correlations.

**Table 2:** Cytotoxic effects of compounds **1**–**5**, expressed as EC_50_ (± SD) values *.

	DHL ** (1)	DHL–Proline (2)	DHL–Piperidine (3)	DHL–Morpholine (4)	DHL–Tyramine (5)
HL-60	14.1	50.2 ± 1.37	96.5 ± 0.11	166 ± 2.33	>160
Kasumi-1	12.9	28.1 ± 0.37	>160	>160	>160
KG-1	18.7	97.7 ± 0.89	182 ± 28.11	129 > 20	>160
MOLM-13	12.6	20.6 ± 0.43	52.3 ± 0.65	>160	>160
MV4-11	5.0	23.3 ± 0.16	66.9 ± 5.10	21.1 ± 1.40	>160
THP-1	16.8	26.7 ± 3.73	59.3 > 20	54.4 ± 0.67	>160
TUR	12.2	35.9 ± 1.49	>160	>160	>160
U937	18.9	33.0 ± 4.87	>160	74.7 ± 9.38	>160
***	13.9	37.9	>90.1	>69.8	>160

* Range tested: 1.25–160 μM. ** Taken from Ordóñez et al. [3]. *** Average EC_50_ values. DHL: dehydroleucodine.

## References

[1] Guzman M.L., Jordan C.T. (2005). Feverfew: Weeding out the root of leukaemia. Expert Opin. Biol. Ther..

[2] Zhang Q., Lu Y., Ding Y., Zhai J., Ji Q., Ma W., Yang M., Fan H., Long J., Tong Z. (2012). Guaianolide sesquiterpene lactones, a source to discover agents that selectively inhibit acute myelogenous leukemia stem and progenitor cells. J. Med. Chem..

[3] Ordóñez P.E., Sharma K.K., Bystrom L.M., Alas M.A., Enriquez R.G., Malagón O., Jones D.E., Guzman M.L., Compadre C.M. (2016). Dehydroleucodine, a Sesquiterpene Lactone from Gynoxys verrucosa, Demonstrates Cytotoxic Activity against Human Leukemia Cells. J. Nat. Prod..

[4] Coricello A., Adams J.D., Lien E.J., Nguyen C., Perri F., Williams T.J., Aiello F. (2018). A Walk in Nature: Sesquiterpene Lactones as Multi-Target Agents Involved in Inflammatory Pathways. Curr. Med. Chem..

[5] Ren Y., Yu J., Douglas Kinghorn A. (2016). Development of Anticancer Agents from Plant-Derived Sesquiterpene Lactones. Curr. Med. Chem..

[6] Woods J.R., Mo H., Bieberich A.A., Alavanja T., Colby D.A. (2013). Amino-derivatives of the sesquiterpene lactone class of natural products as prodrugs. MedChemComm.

[7] Neelakantan S., Nasim S., Guzman M.L., Jordan C.T., Crooks P.A. (2009). Aminoparthenolides as novel anti-leukemic agents: Discovery of the NF-κB inhibitor, DMAPT (LC-1). Bioorg. Med. Chem. Lett..

[8] Lamture G., Crooks P.A., Borrelli M.J. (2018). Actinomycin-D and dimethylamino-parthenolide synergism in treating human pancreatic cancer cells. Drug Dev. Res..

[9] Guzman M.L., Rossi R.M., Neelakantan S., Li X., Corbett C.A., Hassane D.C., Becker M.W., Bennett J.M., Sullivan E., Lachowicz J.L. (2007). An orally bioavailable parthenolide analog selectively eradicates acute myelogenous leukemia stem and progenitor cells. Blood.

[10] Gopal Y.N.V., Arora T.S., Van Dyke M.W. (2007). Parthenolide Specifically Depletes Histone Deacetylase 1 Protein and Induces Cell Death through Ataxia Telangiectasia Mutated. Chem. Biol..

[11] Guzman M.L., Rossi R.M., Karnischky L., Li X., Peterson D.R., Howard D.S., Jordan C.T. (2005). The sesquiterpene lactone parthenolide induces apoptosis of human acute myelogenous leukemia stem and progenitor cells. Blood.

[12] Ghantous A., Gali-Muhtasib H., Vuorela H., Saliba N.A., Darwiche N. (2010). What made sesquiterpene lactones reach cancer clinical trials?. Drug Discov. Today.

[13] Ahn K.S., Aggarwal B.B. (2005). Transcription factor NF-κB: A sensor for smoke and stress signals. Ann. N. Y. Acad. Sci..

[14] Villagomez R., Hatti-Kaul R., Sterner O., Almanza G., Linares-Pastén J.A. (2015). Effect of natural and semisynthetic pseudoguianolides on the stability of NF-κB: DNA complex studied by agarose gel electrophoresis. PLoS ONE.

[15] García-Piñeres A.J., Castro V., Mora G., Schmidt T.J., Strunck E., Pahl H.L., Merfort I. (2001). Cysteine 38 in p65/NF-κB Plays a Crucial Role in DNA Binding Inhibition by Sesquiterpene Lactones. J. Biol. Chem..

[16] Rüngeler P., Castro V., Mora G., Gören N., Vichnewski W., Pahl H.L., Merfort I., Schmidt T.J. (1999). Inhibition of transcription factor NF-κB by sesquiterpene lactones: A proposed molecular mechanism of action. Bioorg. Med. Chem..

[17] Hansch C., Leo A., Mekapati S.B., Kuru A. (2004). QSAR and ADME. Biorg. Med. Chem..

[18] Sheldrick G.M. (2008). A short history of SHELX. Acta Crystallogr. Sect. A Found. Crystallogr..

[19] Livak K.J., Schmittgen T.D. (2001). Analysis of relative gene expression data using real-time quantitative PCR and the 2-ΔΔCT method. Methods.

[20] Parra-Delgado H., Compadre C.M., Ramírez-Apan T., Muñoz-Fambuena M.J., Compadre R.L., Ostrosky-Wegman P., Martínez-Vázquez M. (2006). Synthesis and comparative molecular field analysis (CoMFA) of argentatin B derivatives as growth inhibitors of human cancer cell lines. Bioorg. Med. Chem..

